# *Culex pipiens quinquefasciatus*: a potential vector to transmit Zika virus

**DOI:** 10.1038/emi.2016.102

**Published:** 2016-09-07

**Authors:** Xiao-xia Guo, Chun-xiao Li, Yong-qiang Deng, Dan Xing, Qin-mei Liu, Qun Wu, Ai-juan Sun, Yan-de Dong, Wu-chun Cao, Cheng-feng Qin, Tong-yan Zhao

**Affiliations:** 1 Department of Vector Biology and Control, State Key Laboratory of Pathogen and Biosecurity, Institute of Microbiology and Epidemiology, Beijing 100071, China; 2 Department of Virology, State Key Laboratory of Pathogen and Biosecurity, Institute of Microbiology and Epidemiology, Beijing 100071, China; 3 Department of Epidemiology, State Key Laboratory of Pathogen and Biosecurity, Institute of Microbiology and Epidemiology, Beijing 100071, China

**Keywords:** *Culex pipiens quinquefasciatus*, transmission, vector competence, Zika virus (ZIKV)

## Abstract

Zika virus (ZIKV) has become a threat to global health since the outbreak in Brazil in 2015. Although ZIKV is generally considered an *Aedes-*transmitted pathogen, new evidence has shown that parts of the virus closely resemble *Culex*-transmitted viruses. Therefore, it is important to evaluate the competence of *Culex* species for ZIKV to understand their potential as vectors. In this study, female *Culex pipiens quinquefasciatus* were orally exposed to ZIKV. Mosquito midguts, salivary glands and ovaries were tested for ZIKV to measure infection and dissemination at 2, 4, 6, 8, 12, 16 and 18 days post exposure (pe). In addition, saliva was collected from mosquitoes after infection and infant mice were bitten by infected mosquitoes to measure the transmission ability of *Cx. p. quinquefasciatus*. The results showed that the peak time of virus appearance in the salivary glands was day 8 pe, with 90% infection rate and an estimated virus titer of 3.92±0.49 lg RNA copies/mL. Eight of the nine infant mice had positive brains after being bitten by infected mosquitoes, which meant that *Cx. p. quinquefasciatus* could be infected with and transmit ZIKV following oral infection. These laboratory results clearly demonstrate the potential role of *Cx. p. quinquefasciatus* as a vector of ZIKV in China. Because there are quite different vector management strategies required to control *Aedes (Stegomyia)* species and *Cx. p. quinquefasciatus*, an integrated approach may be required should a Zika epidemic occur.

## INTRODUCTION

Zika virus (ZIKV) is a mosquito-borne flavivirus that was first isolated in rhesus monkeys from Uganda in 1947.^1^ Human infections were initially reported in the 1950s in Africa.^2, 3^ Outbreaks of ZIKV disease occurred in the islands of the Pacific ocean during 2007–2013.^4, 5, 6^ The first ZIKV infection in Brazil was confirmed in May 2015.^7, 8^ Thereafter, ZIKV spread throughout South America and caused unprecedented outbreaks. On 1 February 2016, the World Health Organization declared a public health emergency of international concern owing to the recent cluster of congenital microcephaly cases and other neurological disorders that have been reported in the Americas in association with the ZIKV pandemic. A total of 66 countries and territories with ZIKV transmission have been documented in the world from 1 January 2007 to 20 April 2016 (World Health Organization situation report).

ZIKV has been detected in *Aedes* mosquitoes, including *Ae. africanus*, *Ae. luteocephalus*, *Ae. aegypti* and *Ae. Albopictus*, from Senegal, the Ivory coast, Burkina Faso, the Central African Republic, Uganda and Asia.^1, 9, 10^ *Ae. aegypti*, *Ae. albopictus* and *Ae. hensilli* have been shown to be potential vectors of ZIKV based on experimental infection and transmission.^11, 12, 13, 14, 15, 16^ Although ZIKV is generally considered an *Aedes-*transmitted pathogen,^17^ it belongs to the Spondweni serocomplex, which is close to the Japanese encephalitis virus (JEV) complex according to phylogenetic analysis and E protein structures.^18, 19, 20^ Viruses within the JEV complex are transmitted mostly by *Cx.* mosquitoes, such as *Cx. tritaeniorhynchus*, *Cx. P. quinquefasciatus* and *Cx. P. pipiens*.^21, 22^ Therefore, the competence of *Culex* species in ZIKV transmission should be studied to understand their potential as vectors.

*Cx. p. quinquefasciatus* is a member of *Cx. pipiens* complex and is a known vector of St. Louis encephalitis and West Nile viruses in North America^21^ and a secondary vector of JEV in China.^23, 24^ *Cx. p. quinquefasciatus* is widely distributed south of Yangtze river, where it is the most common mosquito species in urban and rural areas of China.^23^ Compared with *Ae. aegypti* and *Ae. albopictus*, *Cx. p. quinquefasciatus* has much greater densities in human habitats.^24^ Although *Cx. p. quinquefasciatus* is an opportunistic blood feeder, humans are its common host in urban China.^25^ The current paper reports the experimental infection and transmission of ZIKV by *Cx. p. quinquesciatus* mosquitoes.

## MATERIALS AND METHODS

### Ethics statement

All of the experimental protocols involving animals were approved by the Laboratory Animal Center of the State Key Laboratory of Pathogen and Biosecurity, Beijing Institute of Microbiology and Epidemiology Institutional Animal Care and Use Committee (IACUC, the permit number is BIME 2011-2009). The study of animals was performed in strict accordance with the recommendations of the Guide for the Care and Use of Laboratory Animals of the National Institutes of Health.

### Mosquitoes

The *Cx. p. quinquefasciatus* used for experimental infection were collected as larvae from Hainan province of southern China in the summer of 2014 and reared in the laboratory. The mosquitoes were maintained under standard insectary conditions at 26±1 °C, 75±5% relative humidity and a 14 h:10 h light:dark photoperiod. Prior to the infectious feed, adult mosquitoes were provided with 8% sugar water.

### Virus

The mosquitoes were exposed to a contemporary ZIKV strain SZ01 (GenBank NO. KU866423). This virus was originally isolated from the blood of a patient who returned to China from Samoa in 2016.^26^ The stock virus used in the current study has been passaged twice in C6/36 cells prior to the infectious feed.

### Oral infection of mosquitoes

Seven-day-old female mosquitoes (*n*>500) were starved for 12 h prior to the infectious blood meal. The blood meal consisted of 1:1 mouse blood and virus suspension. Oral infections with ZIKV were performed with virus at a titer of 3 × 10^5^ plaque-forming units/mL, verified by titration in a standard plaque assay.^27^ The mosquitoes were fed with an infectious blood meal that was warmed to 37 °C using a Hemotek membrane feeding system housed in a feeding chamber. After 30 min, the mosquitoes were cold anesthetized, and fully engorged females were transferred to 300 mL ca. plastic cups, which were maintained at 29±1 °C, 75±5% relative humidity, a 14 h/10 h light:dark photoperiod and 8% sugar water.

### Mosquito processing

To determine the ZIKV infection and dissemination rates in *Cx. p. quinquefasciatus*, ten female mosquitoes were sampled on days 2, 4, 6, 8, 12, 16 and 18 postexposure (pe). To prevent cross-contamination of the virus across the midgut, salivary glands and ovaies of each mosquito, these organs were carefully dissected using different dissecting needles, and the organs were rinsed in phosphate-buffered saline thrice. The midguts, salivary glands and ovaries from each mosquito then were individually transferred to 1.5-mL microtubes containing 100 mL of Dulbecco's modified Eagle's medium (DMEM; GIBCO, Invitrogen, Beijing, China) supplemented with 2% fetal bovine serum (FBS). These organs were then homogenized using five-mm stainless steel grinding balls (Next Advance, Averill Park, NY, USA) in a Bullet Blender 24 mixer mill (Next Advance) set at a frequency of 12/s for one min. All dissecting needles were dipped in 80% ethanol and burned before being re-used.

### Transmission experiments

To determine the ability of *Cx. p. quinquefasciatus* to transmit ZIKV, on day 8 pe, nine one-day-old infant mice were placed in a cage containing ~100 mosquitoes that previously were fed with the virus–blood mixture described above. After two-h exposure to mosquitoes, the infant mice were removed from the cage and reared separately. In addition, mosquitoes were anesthetized with CO_2_ and 18 blood-engorged females were removed for dissection. The salivary glands of each mosquito were removed and individually transferred to 1.5-mL microtubes containing 100 mL DMEM (GIBCO) supplemented with 2% FBS. On the day 10 postsucking, the nine infant mice were killed, and the brains were excised. In all, 100 mg brain tissue was individually transferred to 1.5-mL microtubes containing 100 mL DMEM (GIBCO) supplemented with 2% FBS.

To determine ZIKV transmission rates, saliva was collected from the individual mosquitoes on days 6, 8, 12 and 16 pe as previously described in Dubrulle *et al.*^28^ Briefly, the wings and legs of each mosquito were removed, and the proboscis was inserted into a quartz capillary containing five μL of FBS. After 45 min, the FBS containing saliva was expelled in 100 μL of DMEM medium for ZIKV detection.

### Detection of the virus

Total RNA was isolated from the midguts, salivary glands, saliva and ovaries of the mosquitoes and the brains of the infant mice using the QIAamp Viral RNA Mini Kit (Qiagen, Hilden, Germany) following the manufacturer's recommendations. ZIKV in these samples was detected using the one-Step Detection Kit for Zika Virus RNA (Daan Gene, Guangzhou, China), which included the reverse primer 5′-TAC AAG TAC CAT CCT GAC TCC C-3′, the forward primer 5′-CYG TCA GTT GYA CTC CAT TCT C-3′ and a LNA-probe (5′-FAM-AGC TCC CCT TCY ACT GAT YTC CAC AT-BHQ1-3′). For the PCR, the following components were used: 5 μL of each RNA sample, 17 μL of PCR Mixture A, and three μL of PCR Mixture B. The final volume was 25 μL. The amplification reactions were performed in a PCR real-time system from Applied Biosystems (Carlsbad, CA, USA; ABI 7500) that was programmed for one cycle at 50 °C for 15 min, 95 °C for 15 min and 40 cycles at 94 °C for 15 s and 55 °C for 45 s.

The number of viral RNA copies was calculated by generating a standard curve from RNA isolated from uninfected mosquitoes, triturated with a known amount of seed virus, the titer of which was determined by plaque assay. The amount of RNA is expressed as lg RNA copies/mL.

### Infection and transmission analysis

The infection rate (IR) of midguts, salivary glands and ovaries at each sampling day was calculated by dividing the number of infected midguts, salivary glands and ovaries by the total number of mosquitoes tested. The transmission was confirmed with appearance of brain-virus-positive in infant mouse brains that were bitten at least once by infected mosquitoes. The transmission rate was calculated by dividing the number of mosquitoes with infected saliva by the number of mosquitoes with a disseminated infection (i.e., midgut-/salivary gland-/ovary-positive). The transmission efficiency represents the proportion of mosquitoes with infectious saliva among the total number of mosquitoes tested.

## RESULTS

### Oral susceptibility of *Cx. p. quinquefasciatus* to ZIKV

**Figure 1:**
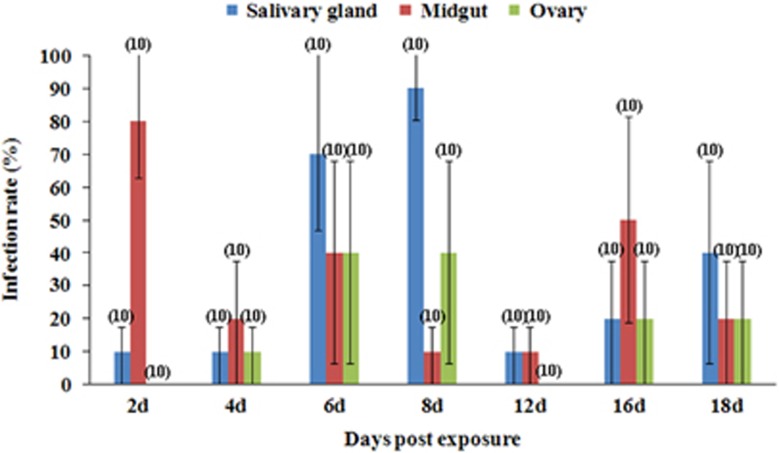
Midgut, salivary gland and ovary ZIKV infection rates in *Cx. p. quinquefasciatus* at different days postexposure to the blood meal. Ten mosquitoes were sampled per day. The error bars represent the confidence intervals (95%). The number of individuals analyzed is given in parentheses. ZIKV, Zika virus.

From day 2 pe onwards, after the blood meal had been completely digested, ZIKV could be detected in the mosquitoes on each of the sampling days (Figure 1). Although the IR of the midgut declined from 80% at the day 2 pe, this IR always remained above 10%. The IR of the salivary glands ascended from 10% on day 2 to 90% on day 8 pe and susequently declined to 10%–40% on days 12–18 pe. The ovary IR was relatively low, peaking at 40% on days 6–8 pe.

### ZIKV titers in the midguts, salivary glands and ovaries of the mosquitoes

Virus titers were calculated by a real-time reverse transcription-PCR and expressed as equivalent lg RNA copies/mL. The standard RNA used in the nucleic amplification assays was extracted from virus dilutions of a known titer, which had been determined by plaque assay.

**Figure 2:**
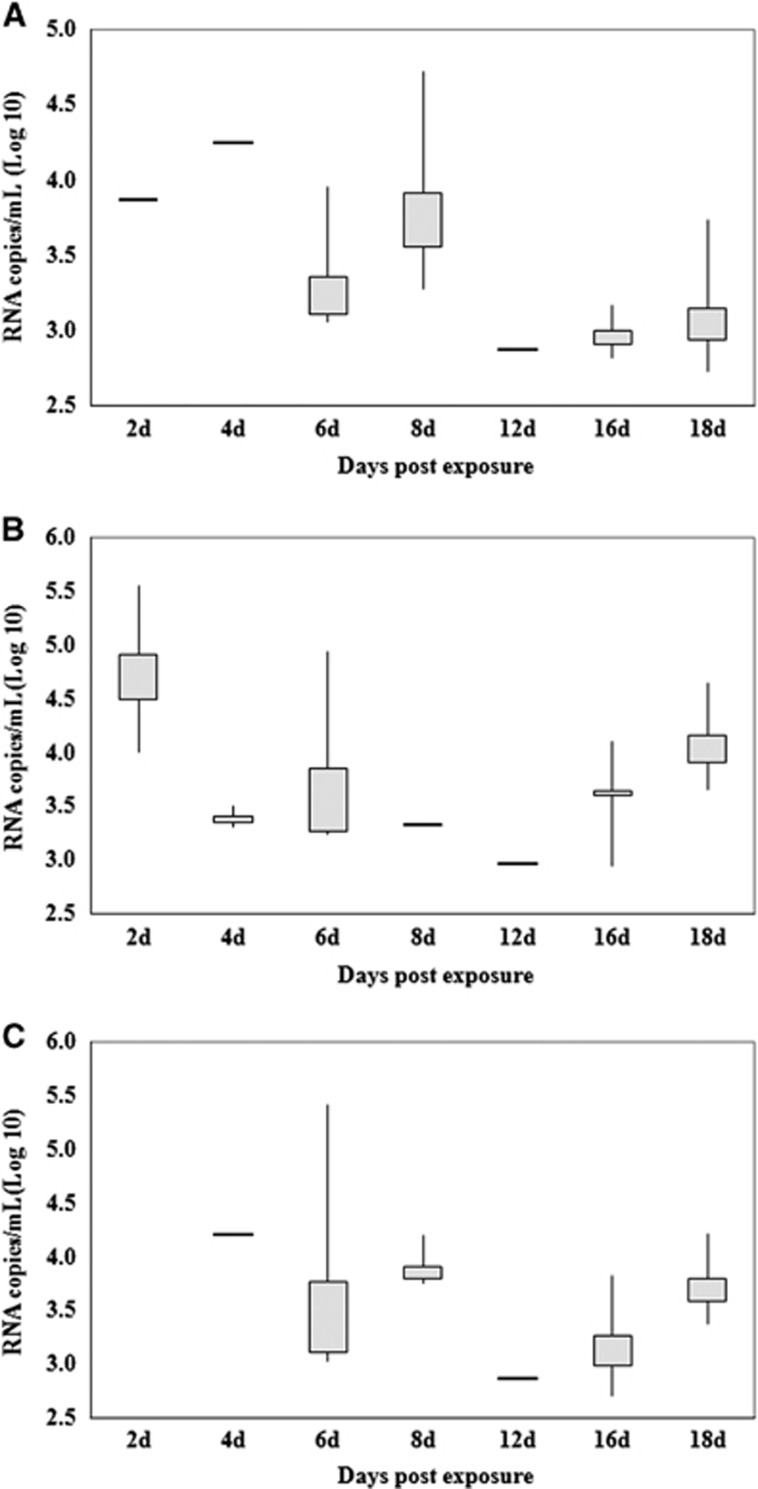
Quantification of Zika viral RNA copies by real-time RT-PCR detected in the salivary glands (**A**), midguts (**B**) and ovaries (**C**) at different days postexposure. Each box represents the median (horizontal bar), the interquartile range (box) and the full range (‘whiskers'). RT-PCR, reverse transcription-PCR.

Figure 2 presents the estimated mean ZIKV titers of the midguts, salivary glands and ovaries of infected females at different days pe. In the midgut, the highest and lowest titers were detected on days 2 and 12 pe, with average values of 4.94±0.97 lg RNA copies/mL (IR=80%) and 2.98 lg RNA copies/mL (IR=10%), respectively. The virus must go through the midgut barrier and spread to the salivary glands and ovaries to cause the infection. Therefore, the times of virus detected in salivary glands and ovaries was later than the time of detection in the midgut.

As illustrated in Figures 1 and 2, the peak time of virus appearance in the salivary glands and ovaries of the mosquitoes was day 8 pe. Not only did the IR of the samples peak at the time (90% and 40%, respectively), but the virus titer was also at a high level (3.92±0.49 and 3.91±0.20 lg RNA copies/mL, respectively). Although the virus titers reached 4.25 and 4.22 lg RNA copies/mL in the salivary glands and ovaries, respectively, at day 4 pe, the infection efficiency of mosquitoes was less on this day than on the other days, because the IR of the salivary glands and ovaries were 10%.

### Transmission of ZIKV by *Cx. p. quinquefasciatus*

ZIKV RNA was detected in the mosquito saliva, which indicated that this virus could be transmitted by mosquitoes. As indicated by the results in Table 1, transmission was first observed on day 8 after the infectious blood meal. At day 6 pe, although the ZIKV was detected in seven of the ten mosquito salivary glands, the viral RNA was not be detected in the saliva. At day 8 pe, RNA was detected in nine of the ten mosquito salivary glands and eight saliva samples were positive with an estimated titer of 4.90±0.56 lg RNA copies/mL. On day 12 pe, only one of the ten mosquitoes was virus RNA-positive in the salivary glands, and the saliva of this mosquito was also virus RNA-positive. However, the estimated virus titer in the saliva was only 3.88 lg RNA copies/mL. On day 16 pe, two of the ten mosquitoes were virus RNA-positive in salivary glands; however, the saliva samples were negative.

The infant mice that has been bitten by infectious mosquitoes can be infected with the virus, and the virus can break through blood–brain barrier and then replicate in the mouse brain, which is direct evidence that this mosquito species can transmit ZIKV and is a potential vector. More than one red blotch was present on the skin of all nine infant mice after removal from the virus-infected mosquito cage, which indicated that all of the infant mice were bitten by at least one mosquito. Among 18 blood-engorged mosquitoes, 15 had viral RNA in their salivary glands. On day 10 pe, eight of the nine infant mice (88.9%) had viral RNA in their brain, with an average estimated virus titer of 7.85±0.76 lg RNA copies/mL.

## DISCUSSION

Zika was previously a neglected tropical disease, and similar to chikungunya fever, interest in ZIKV epidemiology was limited until the outbreak in Brazil in 2015. Currently, Zika is considered a major threat to global health in both the developing and developed countries.^29^ The tendency of these viruses to spread outside their known geographic range and cause large-scale epidemics relies partly on our knowledge of vector distribution. Some mosquito species have been proved to be involved in the transmission cycle of ZIKV, including *Ae. aegypti*, *Ae. albopictus* and some other *Aedes* species.^9, 10, 11, 12, 13, 14, 15^ However, the roles of mosquito species other than those in the genus *Aedes* remain undefined.

*Cx. p. quinquefasciatus* are involved in the transmission of several flaviviruses, such as West Nile and St. Louis encephalitis viruses in the Americas.^21, 22^ In China, *Cx. p. quinquefasciatus* is a secondary vector of JEV^23, 24^ and has been found to be a potential vector of West Nile virus.^30^ In recent research, sequence and structural comparisons of the ZIKV envelope (E) protein with those of other flaviviruses have revealed that parts of the protein closely resemble the neurovirulent West Nile and JEVs, while others are similar to dengue virus.^20^ In this study, we demonstrated that *Cx. p. quinquefasciatus* was able to be infected with and transmit ZIKV after oral exposure. The IR was as high as 90% in the salivary glands and 80% in the saliva on day 8 pe. The decrease in mosquito midgut infections observed over time in our study is consistent with other published studies^31^ and was probably due to virus clearance by the mosquito's immune system.^32, 33^ In the other transmission experiment, infant mice were infected after being bitten by virus-positive mosquitoes and disseminated to and replicated in the mouse brains. These findings verified the potential role of *Cx. p. quinquefasciatus* as a vector of ZIKV.

*Cx. p. quinquefasciatus* is known by the name of ‘southern house mosquito' in China, has a close relationship with humans and inhabits areas around human settlements.^21^ *Cx. p. quinquefasciatus* is the predominant mosquitoes in South China and in other tropical and subtropical areas of the world, especially in urban environments.^34, 35, 36^ Although *Cx. p. quinquefasciatus* feed on both animals and humans, humans are common blood source for this mosquito in urban habitats.^25, 37, 38^ *Cx. p. quinquefasciatus* is the primary species that enters human houses for blood meals during the night in urban areas and towns in China.^25^ There is a lack of animals in urban areas, and therefore *Cx. p. quinquefasciatus* in these areas feed mainly on humans. Even in the places with some animals, humans are an important blood source to *Cx. p. quinquefasciatus*. Although *Cx. p. quinquefasciatus* may not be the primary vector of ZIKV (*Ae. aegypti* occupies that role) owing to its high density, human blood-feeding behavior and vector competence shown herein, its potential importance in the transmission of ZIKV cannot be ignored, especially in urban areas and towns.

This is the first report about the potential of *Cx. p. quinquefasciatus* to transmit ZIKV in China. Given that *Cx. p. quinquefasciatus* is a potential vector of ZIKV, vector control approaches and methods may need to be updated and modified to control ZIKV. Previous vector management strategies have primarily focused on *Aedes* species, similar to dengue vector control programs. There are substantial differences in vector management strategies for *Aedes* species, i.e., *Ae. aegypti* and *Ae. albopictus* and *Cx. p. quinquefasciatus*.^24^ Source reduction needs to target more types of standing water. Additionally, more adult control methods should be implemented to control mosquitoes if a Zika epidemic occurs.

## Figures and Tables

**Table 1:** Transmission rates of ZIKV in ***Cx. p. quinquefasciatus*** on days 6, 8, 12 and 16 postexposure to the blood meal (10 mosquitoes were sampled per day)

**Day pe**	**Number of mosquito tested**	**Number of ZIKV-positive mosquito**^a^	**Number of ZIKV-positive mosquitoes in salivary gland**	**Number of ZIKV-positive mosquitoes in saliva**	**TR of mosquito**^b^	**TE of mosquito**^c^	**ZIKV titer (/g RNA copies/mL) in saliva**
6	10	9	7	0	0.0%	0.0%	—
8	10	10	9	8	80.0%	80.0%	4.90±0.56
12	10	2	1	1	50.0%	10.0%	3.88
16	10	6	2	0	0.0%	0.0%	—

Abbreviations: transmission efficiency, TE; transmission rate, TR; Zika virus, ZIKV.

a Number of ZIKV-positive mosquitoes indicates the number of mosquitoes with infected midguts, salivary glands or ovaries. The virus can be detected in any organ means that this individual is virus positive.

b TR of mosquito is calculated by dividing the number of mosquitoes with infected saliva by the number of mosquitoes with a disseminated infection (i.e., midgut-/salivary gland-/ovary-positive).

c TE of mosquito is calculated by dividing the number of mosquitoes with infected saliva by the total number of mosquitoes tested.
